# Preexisting and inducible endotoxemia as crucial contributors to the severity of COVID-19 outcomes

**DOI:** 10.1371/journal.ppat.1009306

**Published:** 2021-02-18

**Authors:** Ilja L. Kruglikov, Philipp E. Scherer

**Affiliations:** 1 Scientific Department, Wellcomet GmbH, Karlsruhe, Germany; 2 Touchstone Diabetes Center, Department of Internal Medicine, Dallas, Texas, United States of America; 3 Department of Cell Biology, University of Texas Southwestern Medical Center, Dallas, Texas, United States of America; University of Colorado Denver, UNITED STATES

To optimize prevention and control of the Coronavirus Disease 2019 (COVID-19), we need to understand why just a small number of infected individuals develop severe forms of COVID-19. Whereas comorbidities such as obesity, type 2 diabetes (T2D), cardiovascular diseases (CVDs), old age of patients, and their ethnicity are recognized as important contributors toward worse disease outcome [1,2], it is still a matter of discussion what the common critical factors are that directly interact with the Severe Acute Respiratory Syndrome Coronavirus 2 (SARS-CoV-2).

At a first glance, the correlations seem odd, since these comorbidities are not simply connected to a single specific disorder and are not even COVID-19 specific—most of them were reported for other viral infections as well, including the Middle East Respiratory Syndrome (MERS) and H1N1 (swine flu). However, recently, we have argued that all of these comorbidities have something in common and are connected via viral–bacterial interactions, initiated by translocation of bacterial products, such as lipopolysaccharide (LPS), from the gut into circulation [1]. Indeed, increased plasma levels of LPS and LPS-binding protein (LBP) are found in obesity and diabetes, and gut dysbiosis is involved in the pathogenesis of insulin resistance. Low-level inflammation induced by systemic prevalence of bacterial products is involved in vascular abnormalities, and LPS levels in circulation are significantly modified in CVD [3]. Further, the LPS levels in blood are almost doubled in older individuals compared to younger individuals [4]. Finally, the age-adjusted levels of LPS significantly vary in different ethnic groups, being the highest in South Asians. These levels are statistically lower in women than in men in all ethnic groups [5].

Whereas gut microbiota may determine the predisposition of healthy individuals to COVID-19 [6], direct virus–LPS interactions were not considered as a main pathophysiological driver inducing these effects. However, viruses can bind to bacteria or directly to free LPS, thereby enhancing their attachment to ACE2 receptors on the surface of host cells. Such interactions can dramatically increase the viral infectivity and promote the development of hypercytokinemia [1,7]. A formal demonstration that SARS-CoV-2 can directly interact with LPS through its S protein was given in [8]. Whereas neither S protein nor LPS alone causes any activation of the pro-inflammatory nuclear factor kappa B (NF-κB), the combination of S protein with even low levels of LPS dose dependently increases NF-kB activation and the subsequent cytokine response in monocytic cells in vitro [8].

Several clinical reports demonstrate significantly increased levels of endotoxins in the plasma of severely affected COVID-19 patients. LPS levels were investigated in a prospective study on 19 patients with severe pulmonary forms of COVID-19; almost 90% of them had increased LPS levels—40% demonstrated high and 30% demonstrated very high endotoxin levels [9]. Application of mendelian randomization using 6,492 hospitalized cases and over 1 million controls demonstrated that serum LBP levels strongly correlate with the hospitalization rate of COVID-19 patients [10]. The plasma levels of LBP were also investigated in 39 hospitalized patients with severe COVID-19 with significant cardiac pathology; these levels were initially high and remained high during hospitalization [11].

A similar synergistic enhancement of hypercytokinemia was observed in a murine model of infection with influenza virus pH1N1 [12] as well as with the Porcine Respiratory Coronavirus (PRCV) model [13,14]. In both models, combinations of the virus with LPS caused the induction of severe SARS conditions, disproportional (up to 60 times elevated) hypercytokinemia in lungs, and massive death of infected animals, although a single viral or LPS infection at the same doses did not demonstrate such outcomes.

SARS-CoV-2 is not the first viral infection for which severity is connected to the LPS levels in circulation in humans. LPS in circulation was causally connected to systemic immune activation during HIV infections [15]. A similar correlation was found for the dengue virus that annually causes 50 to 100 million infections in tropical and subtropical countries: LPS, LBP, and soluble CD14 levels in plasma of infected individuals are significantly higher than in healthy controls, and the absolute LPS levels in plasma strongly correlate with disease severity [16].

The baseline levels of LPS detected in individuals without viral infection but with comorbidities typical for severe COVID-19 are significantly lower than the LPS values detected in circulation of severe COVID-19 patients. This suggests that SARS-CoV-2 infection can induce an enhanced endotoxemia in compromised individuals. On the other hand, a “leaky gut” condition can be induced by SARS-CoV-2 infection in seemingly non-compromised individuals as well. Thus, we have to differentiate between the “preexisting” and “induced” endotoxemia in COVID-19 patients. Whereas preexisting endotoxemia in groups at risk for COVID-19 has been intensely studied, the overall influence and degree of induced endotoxemia, especially in connection to SARS-CoV-2, has not yet been properly investigated. The main question here to be addressed is whether SARS-CoV-2 can effectively increase the permeability of the intestinal barrier for bacterial products.

SARS-CoV-2 binds to the ACE2 receptor and induces endocytosis, leading to internalization of the virus/ACE2 complex and thereby effectively triggering a down-regulation of cell surface ACE2 content. ACE2 is widely expressed in many different types of cells, among them cells in lungs, adipose tissue, and small intestinal enterocytes. These intestinal enterocytes can thus extensively interact with SARS-CoV-2, demonstrating intestinal involvement. Relevant to this discussion, SARS-CoV-2 RNA is found in stool samples of more than half of the hospitalized patients [17], and 8% to 13% of COVID-19 patients report diarrhea [18]. At the same time, it is known that ACE2 has at least 2 other functions: negative regulation of the renin–angiotensin system and facilitation of amino acid transport. Amino acids are strongly involved in the regulation of the intestinal epithelial barrier function [19]; hence, a reduction of ACE2 content induced by interactions of these receptors with SARS-CoV-2 will significantly impair the integrity of the intestinal barrier.

The intense discussion of hypercytokinemia in severe COVID-19 was so far mainly focused on the expression of cytokines and almost completely ignored the corresponding feedback mechanisms leading to their suppression. One of these feedback loops connected to LPS is endotoxin tolerance (i.e., reduced cellular responses to repeated applications of endotoxins), which, under quasi-physiological conditions, suppresses the induced endotoxemia. A closer examination of cytokine expression in COVID-19 versus sepsis patients reveals a nonobvious behavior [20]. Whereas a relatively low spontaneous production of some cytokines is observed in monocytes of COVID-19 patients with hyper-inflammation versus controls and in patients with sepsis, additional LPS stimulation caused a strong further enhancement of expression of all cytokines (especially of interleukin [IL]-1β) in COVID-19, but not in septic patients. This observation raises the question whether the “endotoxin tolerance” may be weakened in severely ill COVID-19 patients, thereby explaining the massive hypercytokinemia that does not occur to the same extent in septic patients.

Endotoxin tolerance is an adaptive cellular response connected to suppression of the Toll-like receptor 4 (TLR4). TLR4 is widely expressed in high-risk groups due to preexisting endotoxemia. Upon infection with SARS-CoV-2, these individuals frequently demonstrate hypercytokinemia. This suggests that the development of endotoxin tolerance is impaired by SARS-CoV-2. Indeed, it was reported that ACE2 exhibits a protective effect against LPS-induced acute lung injury in mice [21]; hence, viral suppression of ACE2 can lead to a stronger inflammatory responses in lungs.

Endotoxin tolerance is mechanistically connected to an up-regulation of the protein SRC homology 2 domain-containing inositol-5-phosphatase 1 (SHIP1), whose deficiency makes individuals much more susceptible to LPS [22]. LPS up-regulates SHIP1, thereby inducing a compensatory response by suppression of TLR4 [22]. Important in this context, miR-155, whose expression was recently associated with the development of COVID-19 symptoms [23], is an established potent repressor of SHIP1 [24]. This microRNA is up-regulated during inflammation, is crucial for the development of fibrosis in different tissues, and can also serve as an oncogenic factor. MiR-155 levels are responsive to LPS with a short-term elevation followed by a reduction to baseline during the development of endotoxin tolerance [25]. Some viruses stimulate SHIP1 expression [26]. In contrast, the miR-155-3p and miR-155-5p isoforms are found to be up-regulated in SARS-CoV/CoV-2 infected human lung cells, by up to 16-fold and 3-fold, respectively [27]. Overexpression of miR-155-3p is also seen in lung epithelia of COVID-19 patients [28]. Direct effects of SARS-CoV-2 on SHIP1 expression have not yet been investigated in detail. However, based on the abovementioned experimental results, it is most likely that SARS-CoV/CoV-2 induces a response that is opposite to that seen with other viruses and leads to a suppression of SHIP1. Such a response will deteriorate the compensatory effects on endotoxin tolerance and will contribute toward the disproportionate “cytokine storm” typical for SARS-CoV-2.

COVID-19 in its severe forms is unquestionably a systemic disease. Whereas the main signs of severity in this disease are associated with the development of systemic hypercytokinemia and pulmonary complications, we suspect that more severe outcomes of COVID-19 must be connected with a baseline predisposition toward a compromised state of the intestine (preexisting endotoxemia). Upon infection, a dramatic worsening of this state ensues, primarily through a SARS-CoV-2-induced ACE2 deficiency leading to induced endotoxemia and a deteriorated endotoxin tolerance. The unique role that preexisting endotoxemia may play in the development of severe COVID-19 leads us to postulate that standard measurements of LPS and LBP in plasma, immediately upon receiving a positive COVID-19 test, could be a valuable diagnostic help to identify individuals at risk of severe outcomes. This suggested that pathophysiology can also direct us toward appropriate choices to intervene in this process, particularly toward approaches that aim to bind and clear endotoxins from circulation. These could be high-density lipoprotein (HDL) infusions and/or application of the agonists of peroxisome proliferator-activated receptor gamma (thiazolidinediones) that are widely used in antidiabetic therapy, compounds that can effectively reduce LPS in plasma and dramatically decrease endotoxin-induced cytokines [29,30]. Restoration of endotoxin tolerance through application of known suppressors for miR-155 or stimulators for SHIP1 can be another interesting pathway to reduce hypercytokinemia. Future experimental and clinical research will be needed to test the overall efficacy of these approaches. Furthermore, the pathophysiology proposed here may also suggest preemptive approaches aimed to improve the preexisting endotoxemia in healthy individuals and patients from groups at risk for severe outcomes.
